# Molecular Characterization of Monocyte Subsets Reveals Specific and Distinctive Molecular Signatures Associated With Cardiovascular Disease in Rheumatoid Arthritis

**DOI:** 10.3389/fimmu.2019.01111

**Published:** 2019-05-21

**Authors:** Patricia Ruiz-Limon, Rafaela Ortega-Castro, Nuria Barbarroja, Carlos Perez-Sanchez, Christophe Jamin, Alejandra Maria Patiño-Trives, Maria Luque-Tevar, Alejandro Ibáñez-Costa, Laura Perez-Sanchez, Iván Arias de la Rosa, MaCarmen Abalos-Aguilera, Yolanda Jimenez-Gomez, Jerusalem Calvo-Gutierrez, Pilar Font, Alejandro Escudero-Contreras, Marta E. Alarcon-Riquelme, Eduardo Collantes-Estevez, Chary López-Pedrera, the PRECISESADS Clinical Consortium and Flow Cytometry Study Group, C Marañón

**Affiliations:** ^1^Biomedical Research Institute (IBIMA), Service of Endocrinology and Nutrition, Malaga Hospital Complex (Virgen de la Victoria), Malaga, Spain; ^2^Rheumatology Service, Reina Sofia Hospital, Maimonides Institute for Research in Biomedicine of Cordoba (IMIBIC), University of Cordoba, Cordoba, Spain; ^3^CIBER Fisiopatología de la Obesidad y Nutrición (CIBEROBN), Instituto de Salud Carlos III, Madrid, Spain; ^4^Department of Medicine, School of Clinical Medicine, Addenbroke's Hospital, and Cambridge Institute for Medical Research, University of Cambridge, Cambridge, United Kingdom; ^5^U1227, Université de Brest, Inserm, Labex IGO, CHU de Brest, Brest, France; ^6^Medical Genomics, Center for Genomics and Oncology Research: Pfizer, Andalusian Autonomous Government of Genomics and Oncological Research (GENYO), and University of Granada, Granada, Spain

**Keywords:** rheumatoid arthritis, cardiovascular disease, monocyte subsets, microRNAs, gene profile

## Abstract

**Objectives:** This study, developed within the Innovative Medicines Initiative Joint Undertaking project PRECISESADS framework, aimed at functionally characterize the monocyte subsets in RA patients, and analyze their involvement in the increased CV risk associated with RA.

**Methods:** The frequencies of monocyte subpopulations in the peripheral blood of 140 RA patients and 145 healthy donors (HDs) included in the PRECISESADS study were determined by flow cytometry. A second cohort of 50 RA patients and 30 HDs was included, of which CD14^+^ and CD16^+^ monocyte subpopulations were isolated using immuno-magnetic selection. Their transcriptomic profiles (mRNA and microRNA), proinflammatory patterns and activated pathways were evaluated and related to clinical features and CV risk. Mechanistic *in vitro* analyses were further performed.

**Results:** CD14^++^CD16^+^ intermediate monocytes were extended in both cohorts of RA patients. Their increased frequency was associated with the positivity for autoantibodies, disease duration, inflammation, endothelial dysfunction and the presence of atheroma plaques, as well as with the CV risk score. CD14^+^ and CD16^+^ monocyte subsets showed distinctive and specific mRNA and microRNA profiles, along with specific intracellular signaling activation, indicating different functionalities. Moreover, that specific molecular profiles were interrelated and associated to atherosclerosis development and increased CV risk in RA patients. *In vitro*, RA serum promoted differentiation of CD14^+^CD16^−^ to CD14^++^CD16^+^ monocytes. Co-culture with RA-isolated monocyte subsets induced differential activation of endothelial cells.

**Conclusions:** Our overall data suggest that the generation of inflammatory monocytes is associated to the autoimmune/inflammatory response that mediates RA. These monocyte subsets, -which display specific and distinctive molecular signatures- might promote endothelial dysfunction and in turn, the progression of atherosclerosis through a finely regulated process driving CVD development in RA.

## Introduction

Rheumatoid arthritis (RA) is a systemic autoimmune disease encompassing a complex onset mechanism and a number of associated complications. Cardiovascular disease (CVD) significantly contributes to morbidity and mortality in these patients, promoting up to 50% of deaths (1) and atherosclerosis at the onset of the disease is considered a potential preclinical manifestation. Actually, the risk of CVD events is augmented in the 2 years preceding RA diagnosis (2) and once the disease is diagnosed, the risks of CVD events rise with the development of RA. Increased intima media thickness (IMT) of the common carotid artery is a key indicator of early vascular damage in the process of atherosclerosis (3, 4). RA patients have signs of subclinical atherosclerosis, revealed by increased carotid IMT (5, 6). Moreover, increased IMT has been demonstrated in patients without overt CVD to herald enlarged risk for cardiac events and stroke (7). Mechanisms underlying early atherosclerosis in RA are not well comprehended, but a role of immune cells, inflammation, and autoimmunity in this process has been suggested.

Monocytes/macrophages play a relevant role in the pathogenesis of RA. Infiltration of monocytes into joints promote inflammation and proliferation of the synovium and joint destruction in both the acute and the chronic phase of RA (8). Besides, monocytes secrete proinflammatory cytokines such as IL-1ß, IL-6, and TNF, which are released into the systemic circulation, promoting the endothelial activation/dysfunction that heralds the development of atherosclerosis (9, 10). Monocytes express specific ß2-integrins (i.e., CD11c/CD18, CD11b/CD18) whose ligands [intercellular adhesion molecule-1 (ICAM-1), vascular cell adhesion molecule-1 (VCAM-1)] are expressed on the cell-surface of the activated endothelium (11, 12). Increased integrin overexpression by monocytes facilitates their adherence to activated endothelium and their migration into the arterial wall, where monocyte-derived macrophages differentiate into foam cells and form fatty streaks.

Human monocytes encompass two major subpopulations, the classical (CD14^++^CD16^−^) monocytes (accounting for up to 90% of blood monocytes), and CD16^+^ monocytes, which are subdivided in two subsets: intermediate (CD14^++^CD16^+^) and non-classical (CD14^+^CD16^++^) monocytes. Moreover, it has been demonstrated that an evolving relationship exist between monocyte subsets –from classical, via intermediate to non-classical (13).

The CD14^++^/CD16^+^ “intermediate” monocyte subpopulation contains the majority of interleukin-10 (IL-10)-producing cells (14), and yields high levels of proinflammatory cytokines such as tumor necrosis factor (TNF) and IL-1ß. Their involvement in inflammatory immune responses is further indicated by the increased cell-surface expression of HLA-DR and CCR5 on these monocytes.

In contrast, CD14^+^/CD16^++^ “non-classical” monocytes exhibit high migratory but only limited phagocytic potential (15).

Increased frequency of CD16^+^ monocytes has been reported in patients with active RA (16). Rossol et al. also recognized the existence of an expanded intermediate monocyte population in RA patients without rising of the non-classical monocytes population (17). That study further showed that the frequency of the CD14^++^CD16^+^ monocyte subpopulation in RA promoted a proinflammatory cytokine milieu that induced the generation and maintenance of Th17 cells, which are deeply involved in the pathogenesis of autoimmunity.

In addition, it has been shown that a higher number of total circulating monocytes, and higher numbers of CD14^++^CD16^−^ and CD14^++^CD16^+^ monocytes subsets, predict a reduced clinical response to methotrexate (MTX) in RA patients (18), thus supporting their value in predicting the clinical response to treatment.

Definitely monocytes play a key role in the pathogenesis of both RA and atherosclerosis. Similarly, a recent study demonstrated an association of the elevation of both, specific T cells and intermediate monocyte subpopulations in RA with subclinical coronary artery atherosclerosis (19). However, to date, no study has analyzed the molecular profile of these monocyte subsets and their contribution to the establishment of pro-atherothrombotic status in this autoimmune condition.

This study, developed within the Innovative Medicines Initiative Joint Undertaking (IMI JU) project PRECISESADS framework, was undertook to functionally characterize the CD14^+^ and CD16^+^ monocyte subsets in RA patients, and analyze their involvement in the increased CV risk associated with RA Figure S1.

## Materials and Methods

### Study Design and Participants

One hundred and forty RA consecutive patients and one hundred and forty-five healthy donors (HDs) belonging to the PRECISESADS project –involving 18 recruiting centers from nine countries- were included in this study during a period of 3 years. The experimental protocol was approved by the local ethic committee at each of the intervention centers. All the RA patients fulfilled the American College of Rheumatology/ European League Against Rheumatism criteria for the classification of RA (20). All subjects provided written informed consent.

A second cohort of fifty RA patients and thirty-three HDs recruited at the Department of Rheumatology were included after obtaining approval from the ethics committee of the Reina Sofia Hospital from Cordoba (Spain). None of the HDs had a history of other autoimmune diseases, atherosclerosis, or thrombosis.

Clinical and laboratory parameters of the RA patients and the HD included in the study are displayed in Table 1.

**Table 1:** Clinical details of the Rheumatoid Arthritis patients and healthy donors.

	**RA patients**	**Healthy donors**	***p*-value**
**1st cohort (PRECISESADS)**	*n* = 140	*n* = 145	
**Clinical parameters**			
Women/men, n/n	103/37	112/34	0.539
Age, y	56.66 ± 12.68	46.29 ± 13.25	< 0.001
Evolution time, y	13.42 ± 10.20	…	
RF positive, (%)	72/140 (51.40%)	…	
ACPAs, (%)	93/140 (66.40%)	…	
Smoker, (%)	33/140 (23.57%)	10/140 (6.89%)	0.000
**Laboratory parameters**			
CRP, mg/L	5.96 ± 17.9	1.49 ± 1.9	0.004
**Treatments**			
Corticosteroids, (%)	60/140 (42.90%)	…	
Anti-malarials, (%)	15/140 (10.70%)	…	
Immunosuppressants, (%)	108/140 (77.10%)	…	
Biologics, (%)	74/140 (52.90%)	…	
**2nd cohort**	*n* = 50	*n* = 33	
**Clinical parameters**			
Women/men, n/n	30/20	24/9	0.263
Age, y	51.55 ± 11.53	46.70 ± 9.03	0.051
Evolution time, y	5.91 ± 6.1	…	
Pathological CMIT	11/28 (39.28%)	0/7 (0.00%)	0.032
RF positive, (%)	28/46 (60.86%)	…	
ACPAs, (%)	38/45 (84.44%)	…	
DAS28	3.18 ± 1.19	…	
Hypertension, (%)	9/43 (20.93%)	1/33 (3.00%)	0.090
Smoker, (%)	11/43 (25.58%)	5/33 (15.15%)	0.508
**Laboratory parameters**			
CRP, mg/L	7.41 ± 11.02	1.27 ± 1.88	0.001
ESR, mm/h	19.20 ± 13.44	7.8 ± 4.56	< 0.001
**Treatments**			
Corticosteroids, (%)	20/34 (58.82%)	…	
Anti-malarials, (%)	12/31 (38.70%)	…	
NSAIDS, (%)	30/34 (88.20%)	…	
Immunosuppressants, (%)	20/34 (58.82%)	…	
Biologics, (%)	…	…	

*Values are means ± DESVEST. ACPAs, Antibodies to citrullinated protein antigens; RF, Rheumatoid factor; CRP, C reactive protein; ESR, Erythrocyte sedimentation rate; NSAIDS, non-steroidal anti-inflammatory drugs; DAS28, Disease activity score 28. Significant differences vs. healthy donors (p < 0.05)*.

### B-Mode Ultrasound IMT Measurements

All patients and controls underwent B-mode ultrasound imaging for CIMT (carotid intimate media thickness) measurements as previously described (21).

### Endothelial Function: Laser Doppler Linear Periflux 5010

Microvascular function was studied by laser doppler flowmetry as previously reported (22), analyzing the response to reactive hyperemia, so as the increase in blood flow occurred after temporary occlusion of blood flow, using a skin probe attached to the inner forearm.

Post occlusive reactive hyperemia test consisted of 2 min of baseline followed by a 4-min occlusion period. The cuff was then released, and the post occlusive reactive hyperemia response was analyzed for 3 min.

Several parameters were obtained: rest flow (RF), highest perfusion value after occlusion was released (PF), and hyperemic area (HA).

### Monocyte Characterization Through Flow Cytometry. Percentage of Classical, Intermediate and Non-classical Monocytes

Percentage of classical, intermediate and non-classical monocytes PRECISESADs cohort: Whole peripheral blood from RA patients and HD (50 μl) was incubated with PB anti-human CD4, PCy7 anti-human CD14 and FITC anti-human CD16 (Beckman Coulter). Then, blood was lysated and fixated with a mix of both, lysis and fixative solutions (Beckman Coulter) for 20 min at room temperature. Cells were acquired by the 11 different cytometers belonging to each center responsible for the cytometry acquisition: a Navios flow cytometer (Beckman Coulter) was used in three centers, a Gallios (Beckman Coulter) in one center, a FACS Canto II (BD Biosciences) in four centers and a FACS Aria III, a FACS Verse and a LSR Fortessa (BD Biosciences) in one center each. Analysis was performed using an effective multi-center harmonization strategy (22).

2° cohort: Whole peripheral blood from RA patients and HDs (100 μl) was incubated with PB-anti-human CD4, ECD anti-human CD14 (Beckman Coulter, Indianapolis, IN, USA) and APC/Cy7 anti-human CD16 (BioLegend, San Diego, CA, USA) for 20 min at 4°C in the dark. Then, 2 ml of lysis buffer (VersaLyse, Beckman Coulter) was added and incubated 10 min at room temperature. Cells were fixed and acquired on the flow cytometer FC 500 (Beckman Coulter).

Gating strategy: Four-biparametric plots to exclude the doublets were firstly performed. A region including alive cells was created through Draq-7 (Biostatus, Shephed, UK) staining. Using CD4/SSC-A, monocytes population was selected. Finally, from this monocyte population, classical, intermediate and non-classical monocytes were determined according to the expression of CD14 and CD16.

### Intracellular Expression of TNFα, IL-6, and IKK

Whole peripheral blood from RA patients and HDs (100 μl) was incubated with ECD anti-human CD14 (Beckman Coulter, Indianapolis, IN, USA) and APC/Cy7 anti-human CD16 (BioLegend, San Diego, CA, USA). After lysis and Fixation/permeabilization (BD Cytofix/Cytoperm™ Fixation/Permeabilization solution Kit with BD GolgiPlug™; BD Biosciences, San Jose, CA, USA), cells were incubated either with PE anti-human TNF-α (Immunostep, Salamanca, Spain) or PE anti-human IL-6 (Immunostep) or primary antibody anti-human IKK (Abcam, Cambridge, UK) for 30 min at 4°C in the dark. Then, for IKK analysis, PE conjugated secondary antibody (Abcam, Cambridge, UK) was added for 30 min at 4°C. IgG isotypes were used as negative controls. Cells were washed and acquired on the flow cytometer FC 500 (Beckman Coulter).

### Expression of TF Surface

Whole peripheral blood from RA patients and healthy donors (100 μl) was lysated with 2 ml of lysis buffer (VersaLyse; Beckman Coulter) for 10 min at room temperature in the dark. Cells were incubated with ECD anti-human CD14 (Beckman Coulter), APC/Cy7 anti-human CD16 (BioLegend) and FITC-Conjugated monoclonal antibody against human tissue factor (Sekisui Diagnostics, LLC Stamford, CT, USA) for 20 min at 4°C in the dark. IgG isotypes were used as negative controls. Cells were washed and acquired on the flow cytometer FC 500 (Beckman Coulter).

### CD14^+^ and CD16^+^ Monocytes Isolation

Peripheral blood mononuclear cells from RA patients belonging to the second cohort were isolated through ficoll density gradient. Briefly, granulocytes and natural killer cells were removed using immunomagnetic labeling. Firstly, CD16^+^ monocytes were isolated through positive selection (using human anti-CD16, CD16^+^ monocyte isolation kit, MiltenyiBiotec, Bergisch Gladbach, Germany). Thereafter, the CD14^+^ monocytes were isolated from the eluted fluid using positive selection (human anti-CD14, CD14 microbeads, MiltenyiBiotec). Purity of each cell fraction isolated was tested by flow cytometry (FC500 Beckman Coulter), using ECD anti-human CD14 (Beckman Coulter), APC/Cy7 anti-human CD16 (BioLegend). Non-specific antibodies conjugated to ECD and APC/Cy7, respectively, were used as negative controls. Purity of isolated cells was always >90%. The percentage of CD14^+^CD16^+^ (intermediate) and CD14-CD16^+^ (non-classical) cells in CD16^+^ isolated fraction was 55.5 ± 6.2% and 40.3 ± 4.7%, respectively Datasheet 1.

### Total RNA Isolation

Total RNA was extracted from CD14^+^ and CD16^+^ monocytes isolated from RA patients and HDs belonging to the second cohort by using a RNA/DNA/protein purification kit following the manufacturer's instructions (Norgen Biotek Corp., ON, Canada). The RNA purity was verified by optical density (OD) absorption ratio OD260/OD280 between 1.8 and 2.0.

### RT^2^ Profiler Atherosclerosis PCR Array

A human atherosclerosis RT^2^ Profiler PCR array (Qiagen, Hilden, Germany) was used to analyze the expression of 84 genes related to atherosclerosis (https://www.qiagen.com/ch/shop/pcr/primer-sets/rt2-profiler-pcr-arrays/?catno=PAHS-038Z#geneglobe). Specifically, 500 ng of total RNA from CD14^+^ and CD16^+^ monocytes from twelve RA samples belonging to the second cohort and twelve HDs were used. Changes of selected genes (selected among the most significantly altered between both cell populations and further including those related to inflammation and CVD) were validated by quantitative real-time RT-PCR using the LightCycler thermal cycler system (Roche Diagnostics, Indianapolis, USA), using GAPDH as housekeeping gene, as described elsewhere (23).

### microRNA Expression Profiling

The NanoString human v2 array, which contains 800 microRNA probes, was used for microRNA expression data generation. Pools with CD14^+^ and CD16^+^ monocytes RNA purified from 5 RA patients belonging to the second cohort and 5 HDs were obtained.

A total of 100 ng RNA input was used per sample and conditions were set according to the manufacturer's recommended protocol (NanoString Technologies; Seattle, WA). Data were normalized by the geometric mean of all microRNAs detected using the nSolver software. Changes of a number of microRNAs (selected using the same criteria used to choose the mRNAs for validation) were validated in the whole second cohort by quantitative real time PCR by using the miRCURY LNATM Universal RT microRNA PCR system, and specific microRNA primer sets from Exiqon Inc. (Woburn, MA, USA), following the manufacturer's instructions.

### Target Gene Prediction and Integrated Analysis by IPA

Pathway analysis and inverse correlations among expression levels of differentially expressed microRNAs and their respective target mRNAs associated to atherosclerosis were analyzed using Ingenuity Pathway Analysis (IPA, Ingenuity Systems, Redwood City, CA, USA; www.ingenuity.com). *In silico* analysis software revealed enrichment for molecular networks and signaling pathways. The right-tailed Fisher's exact test was used to calculate a *P*-value determining the statistical probability that association between a set of molecules and a pathway or function might be due to chance alone. To minimize false positives among significantly enriched functions, a false discovery rate (FDR) < 0,05 (–log *P*-value = 1,33) was used to determine the probability that each biological function assigned to that data set was due to chance alone'.

Additionally, specific targets (experimentally observed and predicted with high bioinformatics confidence) regulated by the differentially expressed microRNAs were also identified by using the different database integrated in IPA software.

### PathScan Intracellular Signaling Protein Array

Ten microgram of total protein, isolated from CD14^+^ and CD16^+^ monocytes of all RA patients of the second cohort, and HD, in 75 μL were subjected to PathScan intracellular signaling array following the manufacture's recommendations (Cell Signaling Technology, Mass). The phosphorylation levels of ERK1/2, STAT1, STAT3, AKT, AMPKa, S6 ribosomal protein, mTOR, HSP27, Bad, P70 S6 Kinase, PRAS40, p53, p38, SAPK/JNK, and GSK-3b and the cleavage of Caspase-3 and PARP were analyzed on the different monocyte subtypes.

### *In vitro* Studies

Isolated CD14^+^ monocytes from HDs were cultured in medium [RPMI 1640 containing 2 mM L-glutamine, 100 U/ml penicillin, 100 mg/ml streptomycin and 250 pg/ml fungizone (BioWhittaker/MA Bioproducts, Walkersville, MD, USA)] at 37°C in a humidified 5% carbon dioxide (CO2) atmosphere and treated with 10% serum from five RA patients [RA patients showed moderate-low activity (DAS28 3.18 ± 1.19), taking synthetic DMARDs, and not having any biologic DMARDs. They all were female, between 36 and 69 years old, with CRP range from 5 to 15 mg/ml and ESR range from 7 to 30 mm/h positive for ACPAS] or five healthy donors for 96 h. Every 48 h, 400 μl medium was removed from each well and replaced with 500 μl fresh medium containing 10% of RA or HD serum. After 6 and 96 h, cytometer analysis was used to assess phenotypic characterization (FC500 Beckman Coulter).

### Cocultures of RA Monocyte Subtypes-HUVEC

Human umbilical vein endothelial cells (HUVEC) were cultured in Endothelial Cell Basal medium (EBM; Lonza, Walkersville, MD) with 10% FBS, 0.1% human epidermal growth factor (hEGF), 0.1% hydrocortisone, 0.1% gentamicin, amphotericin-B (GA-1,000), 0.4% bovine brain extract, 100 U/ml penicillin, 100 mg/ml streptomycin, and 250 pg/ml fungizone (BioWhittaker/MA Bioproducts, Walkersville, Md) at 37°C in a humidified 5% CO_2_ atmosphere. 10^5^ cells of each, CD14^++^CD16^−^, CD14^++^CD16^+^, and CD14^+^CD16^++^ monocyte populations isolated from 5 RA patients belonging to the second cohort and showing moderate-high activity using cell sorting (FACS ARIA III, Beckton and Dickinson), were seeded into transwell inserts (Corning Transwell polycarbonate membrane cell culture inserts, pore size 0,4 um, Sigma-Aldrich, Mo) in EBM Endothelial Cell Basal medium, and added into multiple plate wells preloaded with HUVEC. Thus, HUVEC and monocytes shared the same culture medium but were physically separated. After 24 h, HUVECs were harvested separately for total RNA isolation and applied to subsequent RT-PCR.

### Statistical Analysis

Statistical analysis used SPSS statistical software, version 19.0 for WINDOWS (SPSS Inc., Chicago, IL, USA). Data are expressed as mean ± SEM. The normal distribution of variables to characterize differences in the analyzed parameters was assessed using the Kolmogorov-Smirnow test. Comparisons among variables were made by paired Student's tests or alternatively by a non-parametric test (Mann-Whitney rank sum tests). In the case of multiple comparison, a Kruskal-Wallis test followed by a Dunn's multiple comparisons test was performed. Categorical data were analyzed using the c^2^ test or Fisher's Exact Probability Test, as appropriate. A study of the relationship among parameters was also carried out using Spearman's rank correlation. Bonferroni correction was further applied in correlation and multiple comparison analyses. Differences were considered significant at *P* < 0.05.

## Results

### Relationship of the Monocyte Subpopulations in RA Patients With Their Immunologic and Inflammatory Profile, and Association to Endothelial Dysfunction, CIMT and CV Risk Score

**Figure 1:**
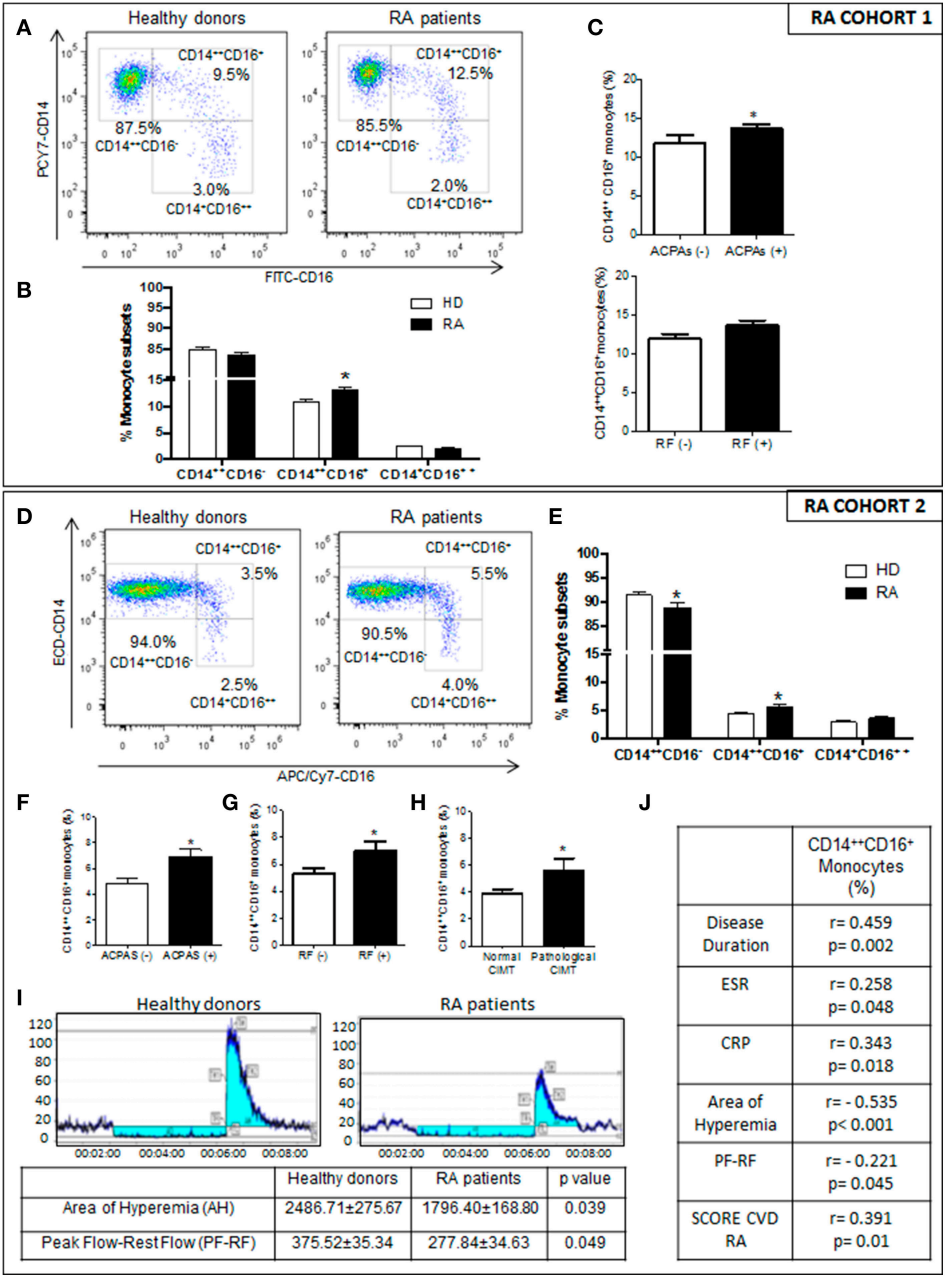
Relationship of the increased intermediate monocyte subpopulation in RA patients with their immunologic and inflammatory profile, and association to endothelial dysfunction, CIMT and CV risk score. **(A–C)** PRECISESADS Cohort. **(A)** Representative dot plots of monocyte subtypes from RA patients and HDs. **(B)** Percentage of different monocyte subtypes by flow cytometry in whole blood of 140 RA patients and 145 HDs. **(C)** Association between increased frequency of intermediate monocytes and autoimmunity. **(D–J)** Second cohort: 50 RA patients and 33 HDs. **(D)** Representative dot plots of monocyte subtypes from RA patients and HDs. **(E)** Percentage of different monocyte subtypes by flow cytometry. **(F–H)** Association between increased frequency of intermediate monocytes with autoimmunity and pathological CIMT. **(I)** Impaired microvascular endothelial dysfunction in RA patients measured by Laser-Doppler. **(J)** Correlations between the percentage of intermediate monocytes and clinical parameters of the disease, endothelial dysfunction and SCORE CVD. Paired *t*-test was performed ^*^indicates significant differences vs. HDs (*p* < 0.05).

CD16^+^ monocytes from patients with RA belonging to the PRECISESADS study could clearly be subdivided into a CD14^++^CD16^+^ intermediate population and a CD14^+^CD16^++^ non-classical population. Only the CD14^++^CD16^+^ population was expanded, while the frequency of CD14^+^CD16^++^ monocytes did not differ between patients and HD (Figures 1A,B). The frequency of the intermediate monocytes was associated with the positivity for ACPAS (Figure 1C). Statistical multivariate analyses further suggested that there were no significant differences in those frequencies among RA patients having diverse therapies, including immunosuppressants, anti-malarials, steroids, and biologics (data not shown).

CD14^++^CD16^+^ monocytes were also significantly extended in RA patients of the second cohort, and associated with the positivity for both, RF and ACPAS (Figures 1D–G). In this cohort, AR patients showed impaired microvascular endothelial function, with a reduced perfusion value after ischemia and hyperemia area (Figure 1I). Increased CD14^++^CD16^+^ frequencies were associated with both, the presence of a pathologic carotid intimae media thickness (CIMT) and microvascular endothelial dysfunction (Figures 1H,J). Besides, the increased percentage of this monocyte subset correlated with the disease duration and the levels of acute phase reactants (CRP and ESR), as well as with the score of CVD in RA patients (Figure 1J).

**Figure 2:**
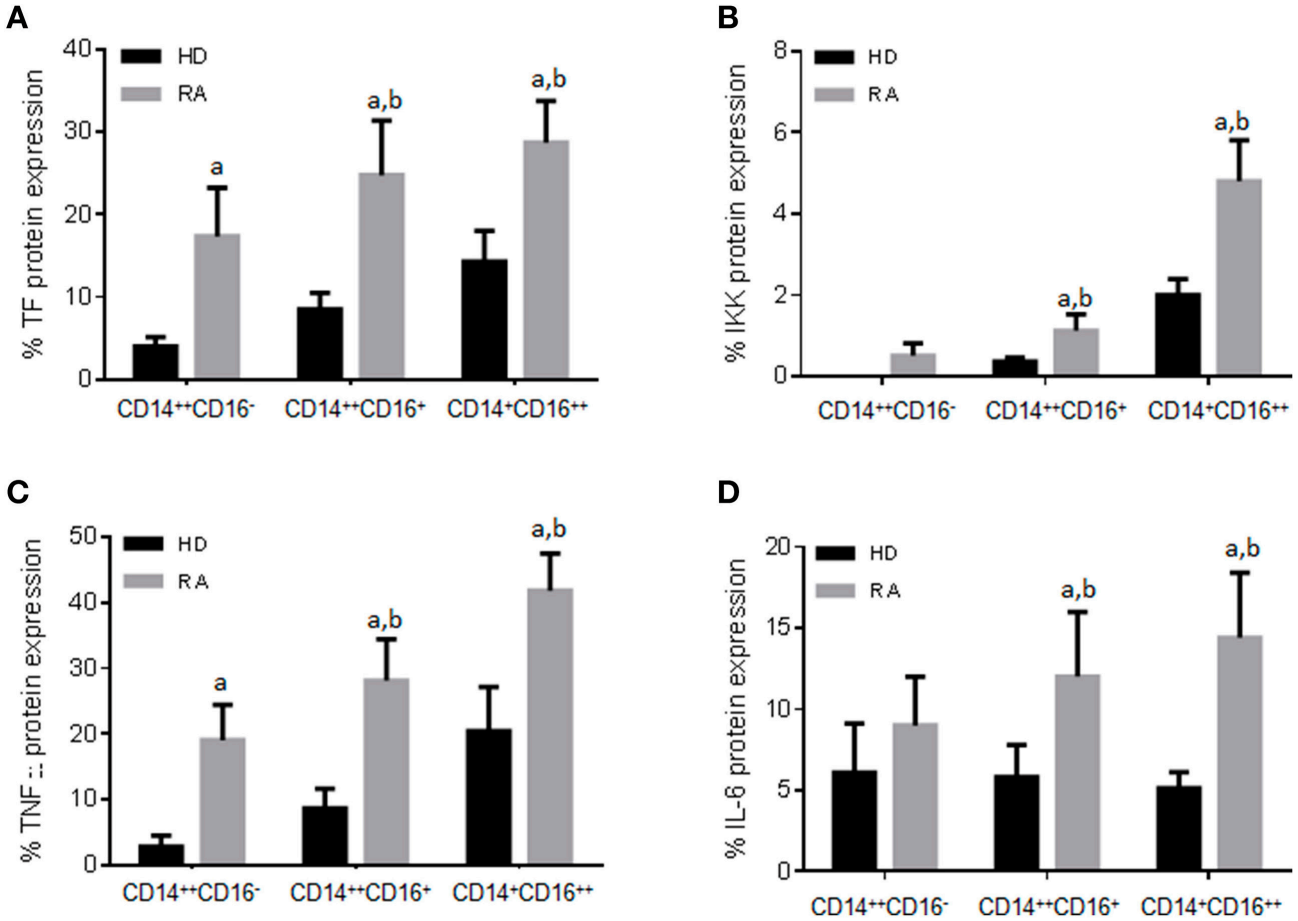
Different protein expression of pro-thrombotic and inflammatory factors in RA monocyte subtypes. **(A–D)** Protein expression of Tissue factor (TF), IκB kinase (IKK), Tumor necrosis factor (TNF) and interleukin 6 (IL-6) in the three subpopulations of monocytes in RA and HDs measured by flow cytometry. Data are presented as mean ± SD, *n* = 50 RA patients and 33 HDs. Paired *t*-test was performed ^a^indicates significant differences vs. HDs, ^b^indicates significant differences vs. CD14^++^CD16^−^ RA monocytes (*p* < 0.05).

Additionally, both intermediate and non-classical monocytes subsets showed higher protein expression of pro-thrombotic [i.e., tissue factor (TF)] and inflammatory factors (i.e., TNF-α, IKK, IL-6) (Figures 2A–D). Percentage of non-classical monocytes was further associated with CV risk factors such as the presence of arterial hypertension (6.1 ± 2.1% on hypertense RA patients vs. 4.0 ± 0.5%; *P* = 0.047) or hypertriglyceridemia (5.9 ± 2.4 vs. 4.0 ± 0.4%; *P* = 0.039).

That overall data demonstrated that both CD16^+^ monocyte subsets shared an altered expression of a number of molecules related to increased CV risk, which might indicate that they also share molecular alterations related to this comorbidity in the setting of RA.

Thus, in order to characterize the molecular profile of monocyte subsets involved in CVD, total CD14^+^ and CD16^+^ monocytes were evaluated.

On the other hand, multiple linear regression analysis was used to determine the association of the percentages of CD14^++^CD16^+^ monocytes with age or the menopausal status and diagnosis (RA vs. HDs). Only diagnosis was statistically proven to act as a confounding variable in the percentage of CD14^++^CD16^+^ monocytes. Indeed, in both RA cohorts, these analyses showed that nor age nor the menopausal status acted as a confounding variable among RA patients in parameters related to the activity of the disease, autoantibody profile (i.e., positivity for RF or ACPAs), and the expression levels of prothrombotic/inflammatory mediators (i.e., TF, IL6 or TNFalpha) (data not shown).

### CD14^+^ and CD16^+^ Monocyte Subsets From RA Patients Show a Specific Gene Expression Profile Related to Atherosclerosis

**Figure 3:**
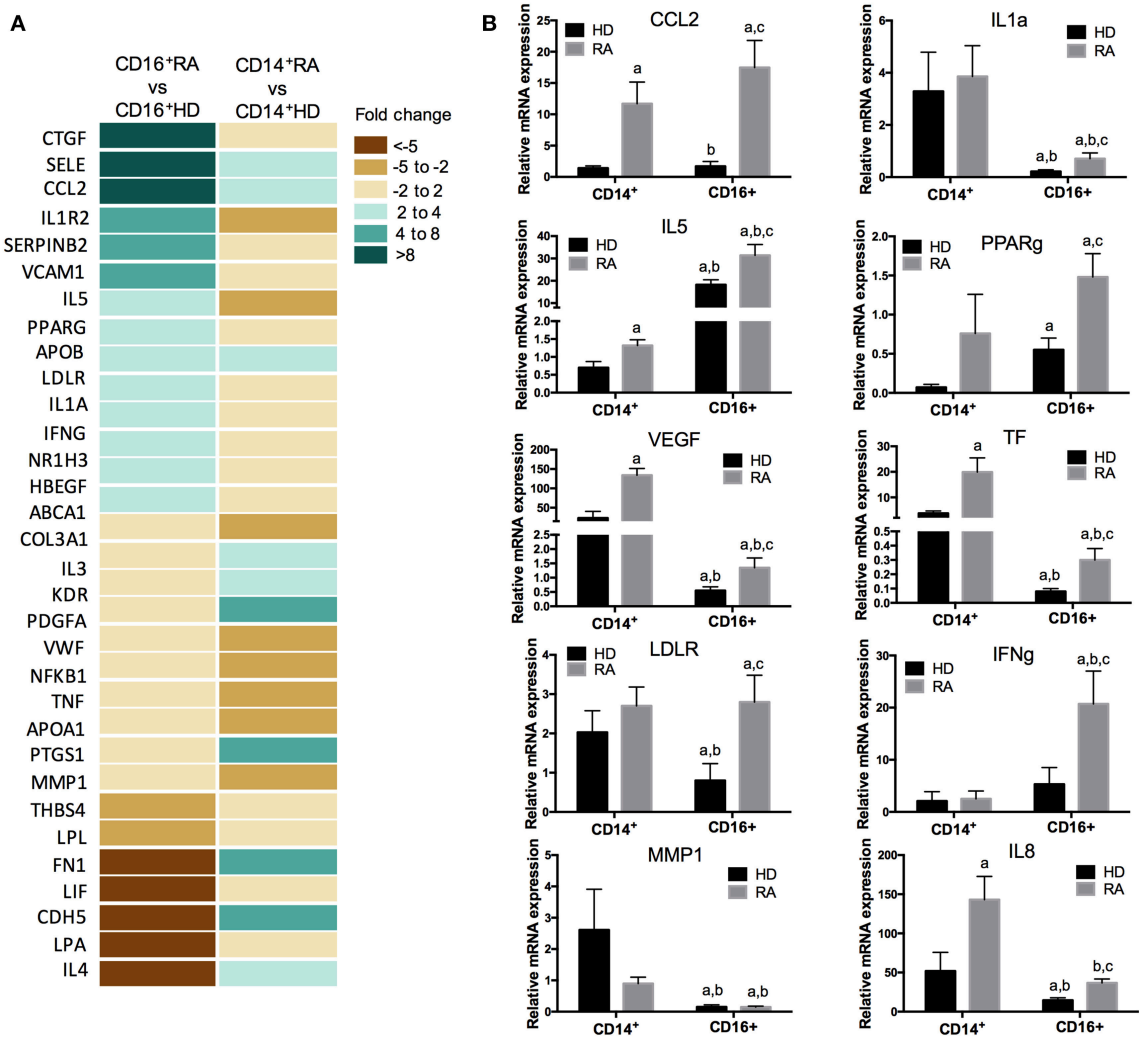
Specific and differential expression profile of genes related to atherosclerosis in CD16^+^ and CD14^+^ monocytes from RA patients compared to HDs. **(A)** Atherosclerosis RT2 profiler PCR array performed in CD14^+^ and CD16^+^ monocytes isolated from 12 RA patients and 12 HDs. Heat-map of the differentially expressed genes in CD16^+^ RA or CD14^+^ RA monocytes vs. CD16^+^ HD and CD14^+^ HD monocytes, respectively. **(B)** Validation of the PCR array in CD14^+^ and CD16^+^ monocytes through RT-PCR in samples of 50 RA patients and 30 HDs, separately. Kruskal-Wallis test, followed by a Dunn's multiple comparison test was performed. ^a^Indicates significant differences vs. CD14^+^ HDs, ^b^indicates significant differences vs. CD14^+^ RA, ^c^indicates significant differences vs. CD16^+^ HDs (*p* < 0.05).

The PCR array showed a specific and differential expression profile of genes related to atherosclerosis in CD16^+^ and CD14^+^ monocytes from RA patients compared to HDs. In CD14^+^ monocytes from RA patients the expression levels of 13 genes were found elevated, whereas 11 genes were found reduced vs. HDs (fold change ≥2; *P* < 0,05). Yet, in CD16^+^ RA monocytes, a distinct set of 14 genes were elevated and 7 genes reduced in relation to HDs (Figure 3A). These altered genes, further validated in the whole cohort of RA patients belonging to the second cohort and HD, were found involved in inflammatory and oxidative stress response (i.e., IL-5, CCL2, PPARγ, and INFγ), adhesion and extracellular signaling (i.e., IL-8 and MMP1), blood coagulation and circulation (i.e., TF and VEGF), lipid metabolism (i.e., LDLR), and cell growth and proliferation (i.e., IL-1α) (Figure 3B).

### CD14^+^ and CD16^+^ Monocytes of RA Patients Displayed Specific and Distinctive microRNA Expression Profiles

**Figure 4:**
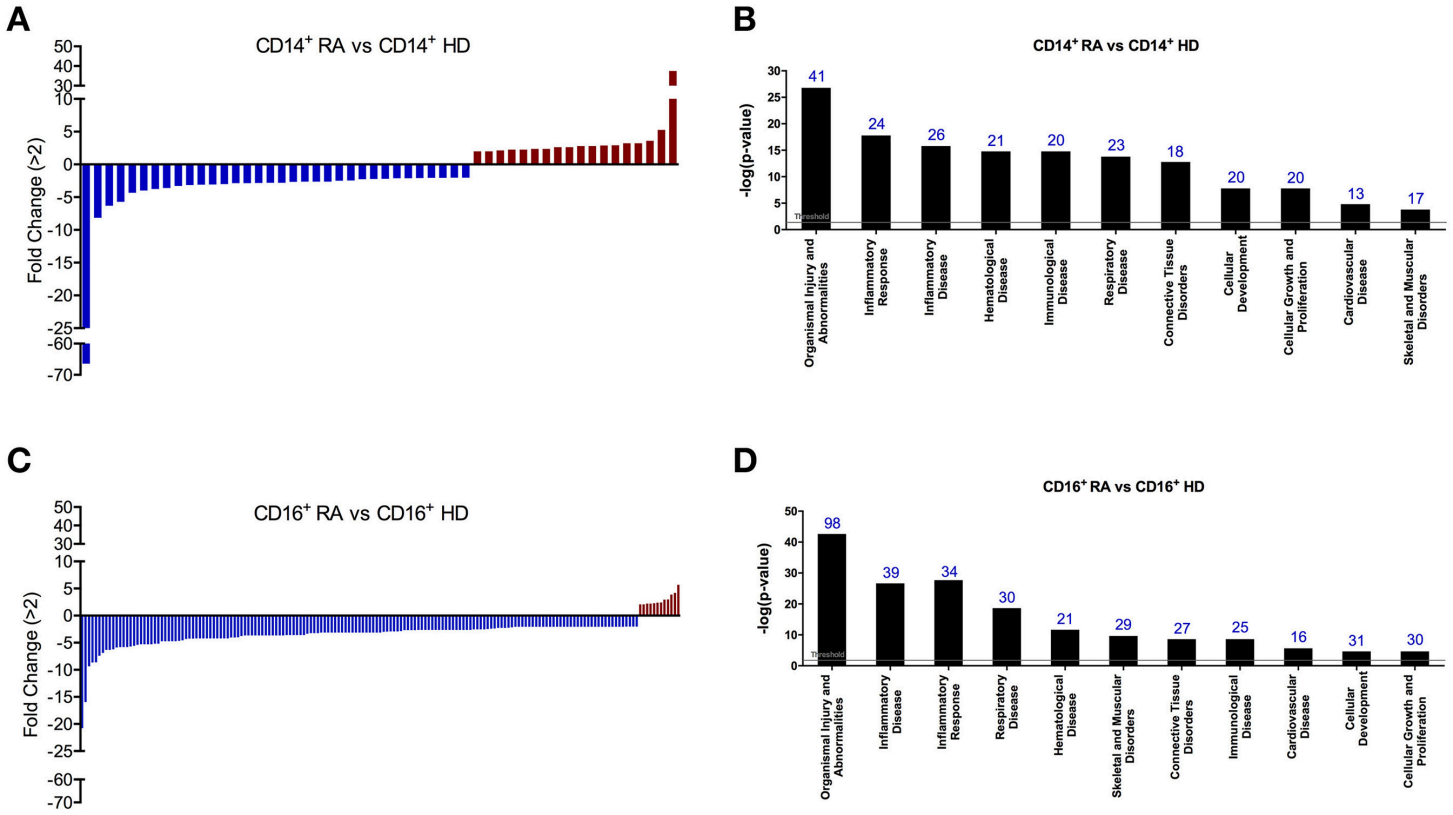
CD14^+^ and CD16^+^ monocytes of RA patients display specific and distinctive microRNA expression profiles. **(A)** Thirty-one microRNAs were found significantly altered in CD14^+^ monocytes isolated from RA patients compared with CD14^+^ HD monocytes using a fold change cut-off of >2. **(B)** Functional classification of the altered microRNAs in CD14^+^ RA monocytes using Ingenuity Pathway Analysis (IPA, QIAGEN Redwood City, https://analysis.ingenuity.com). The analysis included only the functions and pathways with average IPA score >2 [indicated as –log (*p*-value)].To minimize false positives among significantly enriched functions, a false discovery rate (FDR) < 0,05 (–log *P*-value = 1,33) was used to determine the probability that each biological function assigned to that data set was due to chance alone'. Threshold bar indicates cut-off point of significance (*p* > 0.05), using Fisher's exact test, microRNAs. **(C)** One hundred and seventy-three microRNAs were found significantly altered in CD16^+^ monocytes isolated from RA patients compared with CD16^+^ HD monocytes using a fold change cut-off of >2. **(D)** Functional classification of the altered microRNAs in CD16^+^ RA monocytes using Ingenuity Pathway Analysis. The analysis included only the functions and pathways with average IPA score >2 [indicated as –log (*p*-value)]. Threshold bar indicates cut-off point of significance (*p* >0.05), using Fisher's exact test, microRNAs.

**Figure 5:**
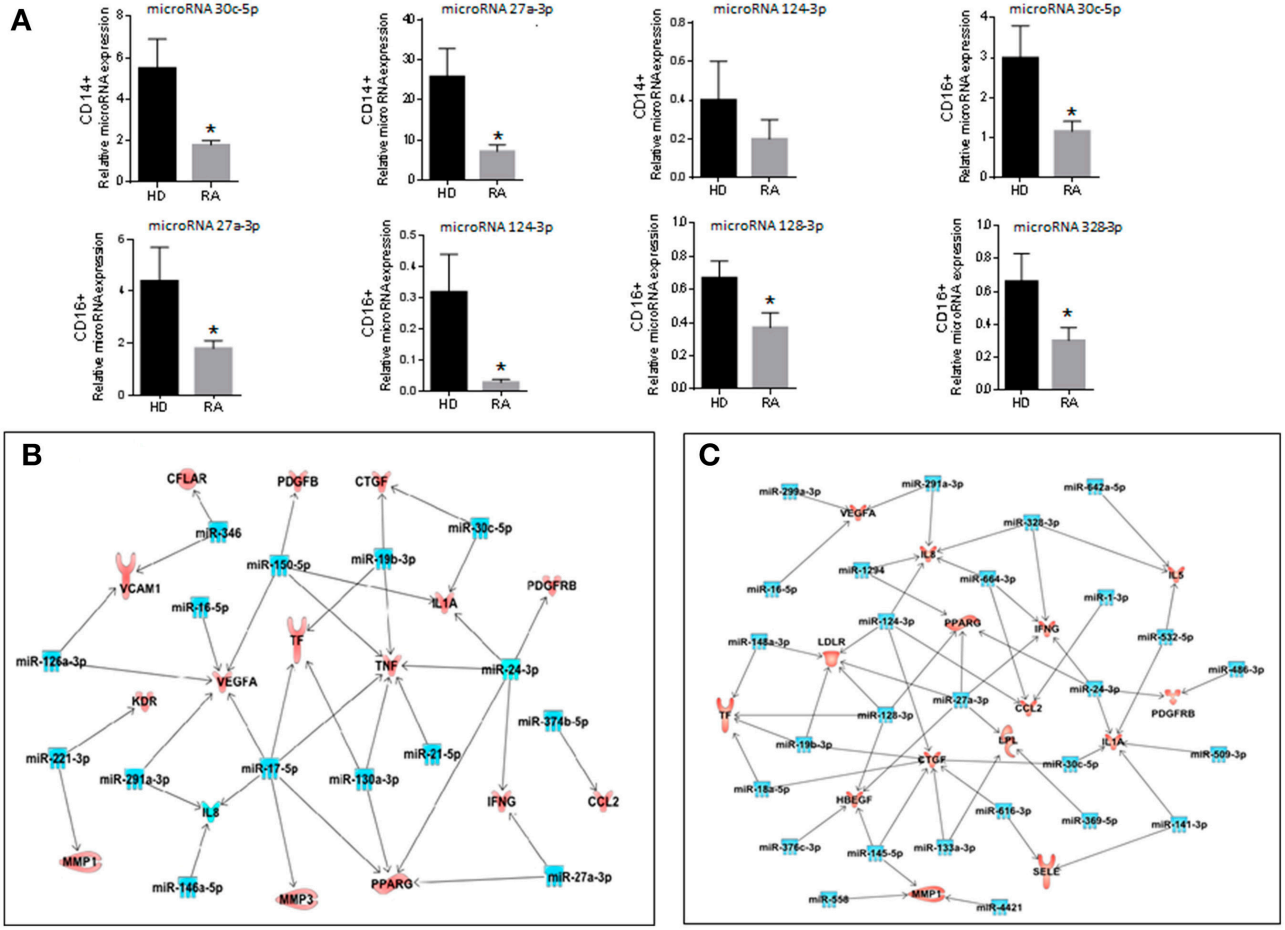
Validation of the microRNA array and integrated analysis between the microRNAs and genes altered in CD14^+^ and CD16^+^ RA monocytes. **(A)** mRNA relative expression of microRNAs in CD14^+^ and CD16^+^ monocytes from RA patients and HDs. Data are presented as mean ± SD, *n* = 50 RA patients and 33 HDs. Paired *t*-test was performed (^*^) indicates significant differences vs. HDs (*p* < 0.05). **(B)** Integrated analysis of altered microRNAs (microRNA array) and mRNA gene expression (PCR array) in CD14^+^ RA monocytes. **(C)** Integrated analysis of altered microRNAs (microRNA array) and mRNA gene expression (PCR array) in CD16^+^ RA monocytes. Interaction networks of altered microRNAs and genes related to atherosclerosis using IPA software.

Differential expression of microRNAs in monocyte subsets of RA patients was determined using nanostring microRNA arrays and subsequent validation by real-time reverse transcription polymerase chain reaction (Figures 4, 5A). Comparing to HDs, 52 microRNAs were found significantly altered in CD14^+^ monocytes from RA patients (cut off ≥2; *P* < 0,05; Figure 4A; Table 2). Functional classification of those microRNAs, using the Ingenuity Pathway Analysis software (IPA), showed a preponderance of target mRNAs involved in organismal injury and abnormalities, inflammatory disease, hematological and immunological disease (Figure 4B).

**Table 2:** MicroRNA expression profile of CD14^+^ monocytes from Rheumatoid Arthritis patients vs. CD14^+^ monocytes from healthy donors.

**CD14^+^ RA vs. CD14^+^ HD**	
**miRNA**	**Fold change**
hsa-miR-4286	−66.44
hsa-miR-516a-5p	−8.14
hsa-miR-199a-5p	−6.32
hsa-miR-146a-5p	−5.69
hsa-miR-126-3p	−4.32
hsa-miR-378e	−3.97
hsa-miR-4516	−3.75
hsa-miR-27b-3p	−3.60
hsa-miR-454-3p	−3.30
hsa-miR-374b-5p	−3.15
hsa-miR-155-5p	−3.08
hsa-miR-221-3p	−3.07
hsa-miR-16-5p	−2.99
hsa-miR-26a-5p	−2.87
hsa-miR-28-3p	−2.87
hsa-miR-324-5p	−2.82
hsa-miR-4454	−2.80
hsa-miR-423-5p	−2.80
hsa-miR-494	−2.67
hsa-miR-423-3p	−2.63
hsa-miR-20a-5p	−2.63
hsa-miR-20b-5p	−2.63
hsa-miR-30c-5p	−2.50
hsa-miR-26b-5p	−2.47
hsa-miR-19b-3p	−2.26
hsa-miR-362-3p	−2.22
hsa-miR-21-5p	−2.19
hsa-miR-24-3p	−2.12
hsa-let-7f-5p	−2.11
hsa-miR-107	−2.07
hsa-miR-106a-5p	−2.05
hsa-miR-17-5p	−2.04
hsa-miR-130b-3p	−2.04
hsa-miR-185-5p	−2.02
hsa-miR-192-5p	2.01
hsa-miR-195-5p	2.01
hsa-miR-216a	2.13
hsa-miR-200b-3p	2.26
hsa-miR-495	2.26
hsa-miR-346	2.38
hsa-miR-485-3p	2.38
hsa-miR-544a	2.62
hsa-miR-761	2.62
hsa-miR-302d-3p	2.80
hsa-miR-363-3p	2.80
hsa-miR-150-5p	2.89
hsa-miR-598	2.93
hsa-miR-188-5p	3.24
hsa-miR-7-5p	3.24
hsa-miR-1183	3.61
hsa-miR-144-3p	5.28
hsa-miR-451a	37.49

**Figure 6:**
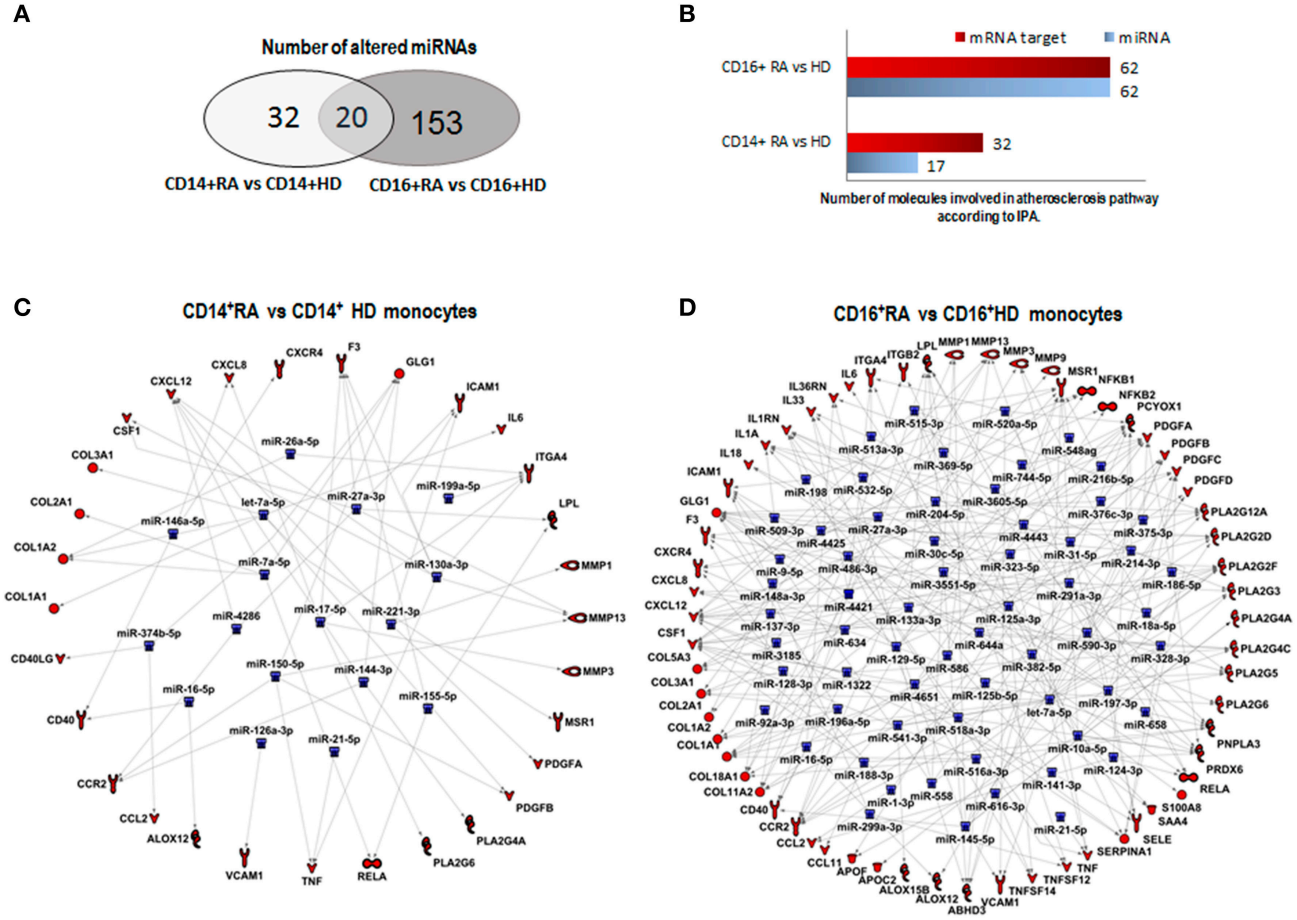
CD16^+^ monocytes exhibit a prevalence of target mRNAs involved in atherosclerosis. **(A)** Number of altered microRNAs in CD14^+^ and CD16^+^ RA monocytes compared to HDs. 31 were specifically regulated in CD14^+^ RA monocytes, 153 were specifically regulated in CD16^+^ RA monocytes and 20 microRNAs were commonly altered in both subtypes in RA patients compared to HDs. **(B)** Among all the altered microRNAs in CD14^+^ and CD16^+^ RA monocytes, number of microRNAs involved in atherosclerosis pathway according to IPA. **(C)** Putative mRNA targets of the microRNAs altered in CD14^+^ RA monocytes compared to CD14^+^ HD monocytes involved in atherosclerosis according to IPA. **(D)** Putative mRNA targets of the microRNAs altered in CD16^+^ RA monocytes compared to CD16^+^ HD monocytes involved in atherosclerosis according to IPA.

A higher number of microRNAs (173) were found altered in CD16^+^ monocytes than in CD14^+^ monocytes of RA patients compared to HDs (cut off ≥2; Figure 4C; Table 3). Among them, 20 microRNAs displayed similar alterations in both monocyte subsets, 32 were specifically modified in CD14^+^ RA monocytes and 153 microRNAs were specifically altered in CD16^+^ (Figure 6A). Functional classification of altered microRNAs in both monocyte subsets displayed a prevalence of target mRNAs involved in organismal injury and abnormalities, inflammatory disease, hematological disease, skeletal, muscular and connective tissue disorders and immunological disease (Figure 4D). Interestingly, IPA analysis demonstrated that the most significantly altered microRNAs in CD16^+^ monocytes exhibited a prevalence of target mRNAs involved in atherosclerosis, while only half of those microRNAs in CD14^+^ monocytes had target mRNAs related to atherosclerosis (Figures 6B–D).

**Table 3:** microRNA expression profile of CD16^+^ monocytes from Rheumatoid Arthritis patients vs. CD16^+^ monocytes from healthy donors.

**CD16**^+^ **RA vs. CD16**^+^ **HD**					
**miRNA**	**Fold change**	**miRNA**	**Fold change**	**miRNA**	**Fold change**
hsa-miR-196b-5p	−20.79	hsa-miR-616-3p	−3.68	hsa-miR-216a	−2.55
hsa-miR-95	−15.97	hsa-miR-890	−3.68	hsa-miR-450b-5p	−2.55
hsa-miR-548ad	−9.37	hsa-miR-378h	−3.61	hsa-miR-28-3p	−2.50
hsa-miR-376c	−8.68	hsa-miR-641	−3.61	hsa-miR-26a-5p	−2.40
hsa-let-7c	−8.62	hsa-miR-1914-5p	−3.60	hsa-miR-129-2-3p	−2.38
hsa-miR-627	−7.42	hsa-miR-124-3p	−3.59	hsa-miR-105-5p	−2.27
hsa-miR-525-5p	−6.89	hsa-miR-302b-3p	−3.59	hsa-miR-372	−2.27
hsa-miR-4458	−6.35	hsa-miR-27a-3p	−3.59	hsa-miR-4421	−2.27
hsa-miR-561-3p	−6.35	hsa-miR-99b-5p	−3.34	hsa-miR-10a-5p	−2.23
hsa-miR-759	−6.23	hsa-miR-30b-5p	−3.26	hsa-miR-423-5p	−2.14
hsa-miR-141-3p	−5.89	hsa-miR-410	−3.26	hsa-miR-1236	−2.08
hsa-miR-18b-5p	−5.82	hsa-miR-423-3p	−3.23	hsa-miR-125a-3p	−2.08
hsa-miR-515-5p	−5.82	hsa-miR-1279	−3.15	hsa-miR-1265	−2.08
hsa-miR-761	−5.82	hsa-miR-1322	−3.15	hsa-miR-1293	−2.08
hsa-miR-758	−5.70	hsa-miR-186-5p	−3.15	hsa-miR-1470	−2.08
hsa-miR-125b-5p	−5.57	hsa-miR-200b-3p	−3.15	hsa-miR-188-3p	−2.08
hsa-miR-195-5p	−5.41	hsa-miR-299-3p	−3.15	hsa-miR-2277-3p	−2.08
hsa-miR-382-5p	−5.29	hsa-miR-299-5p	−3.15	hsa-miR-302f	−2.08
hsa-miR-516a-3p	−5.29	hsa-miR-337-5p	−3.15	hsa-miR-31-5p	−2.08
hsa-miR-524-3p	−5.29	hsa-miR-369-5p	−3.15	hsa-miR-3605-5p	−2.08
hsa-miR-548a-5p	−5.29	hsa-miR-4647	−3.15	hsa-miR-378b	−2.08
hsa-miR-215	−5.18	hsa-miR-519e-3p	−3.15	hsa-miR-431-5p	−2.08
hsa-miR-662	−5.18	hsa-miR-548ai	−3.15	hsa-miR-486-3p	−2.08
hsa-miR-1287	−4.75	hsa-miR-558	−3.15	hsa-miR-490-5p	−2.08
hsa-miR-328	−4.75	hsa-miR-608	−3.15	hsa-miR-509-3p	−2.08
hsa-miR-450b-3p	−4.75	hsa-miR-610	−3.15	hsa-miR-512-3p	−2.08
hsa-miR-550a-5p	−4.75	hsa-miR-887	−3.15	hsa-miR-515-3p	−2.08
hsa-miR-658	−4.75	hsa-miR-935	−3.15	hsa-miR-516a-5p	−2.08
hsa-miR-335-5p	−4.66	hsa-miR-197-3p	−3.14	hsa-miR-526a	−2.08
hsa-miR-590-3p	−4.58	hsa-miR-363-3p	−3.14	hsa-miR-520c-5p	−2.08
hsa-miR-422a	−4.32	hsa-miR-375	−3.01	hsa-miR-518d-5p	−2.08
hsa-miR-302d-3p	−4.24	hsa-miR-532-5p	−2.98	hsa-miR-548i	−2.08
hsa-miR-1183	−4.24	hsa-miR-4443	−2.95	hsa-miR-548l	−2.08
hsa-miR-128	−4.22	hsa-miR-378f	−2.93	hsa-miR-559	−2.08
hsa-miR-133b	−4.22	hsa-miR-590-5p	−2.93	hsa-miR-578	−2.08
hsa-miR-34c-3p	−4.22	hsa-miR-30a-5p	−2.87	hsa-miR-580	−2.08
hsa-miR-487a	−4.22	hsa-miR-145-5p	−2.69	hsa-miR-634	−2.08
hsa-miR-548x-3p	−4.22	hsa-miR-147b	−2.69	hsa-miR-654-3p	−2.08
hsa-miR-566	−4.22	hsa-miR-137	−2.68	hsa-miR-891b	−2.08
hsa-miR-664-3p	−4.22	hsa-miR-485-3p	−2.68	hsa-miR-92a-3p	−2.08
hsa-miR-744-5p	−4.22	hsa-miR-127-5p	−2.61	hsa-miR-92b-3p	−2.08
hsa-miR-885-5p	−4.22	hsa-miR-210	−2.61	hsa-miR-598	−2.06
hsa-miR-934	−4.22	hsa-miR-320a	−2.61	hsa-miR-3185	−2.04
hsa-miR-1	−4.06	hsa-miR-323a-5p	−2.61	hsa-miR-510	−2.04
hsa-miR-376b	−4.06	hsa-miR-4425	−2.61	hsa-miR-30c-5p	−2.04
hsa-miR-192-5p	−4.00	hsa-miR-4532	−2.61	hsa-miR-424-5p	−2.03
hsa-miR-2117	−3.78	hsa-miR-491-5p	−2.61	hsa-miR-500a-5p	2.07
hsa-miR-1294	−3.68	hsa-miR-518a-3p	−2.61	hsa-miR-501-5p	2.07
hsa-miR-129-5p	−3.68	hsa-miR-520b	−2.61	hsa-miR-660-5p	2.20
hsa-miR-196a-5p	−3.68	hsa-miR-541-3p	−2.61	hsa-miR-18a-5p	2.22
hsa-miR-198	−3.68	hsa-miR-548am-3p	−2.61	hsa-miR-19b-3p	2.29
hsa-miR-211-5p	−3.68	hsa-miR-586	−2.61	hsa-miR-642a-5p	2.37
hsa-miR-214-3p	−3.68	hsa-miR-644a	−2.61	hsa-miR-346	2.41
hsa-miR-217	−3.68	hsa-miR-888-5p	−2.61	hsa-miR-450a-5p	2.92
hsa-miR-412	−3.68	hsa-miR-933	−2.61	hsa-miR-324-5p	2.97
hsa-miR-513a-3p	−3.68	hsa-miR-938	−2.61	hsa-miR-148a-3p	3.88
hsa-miR-544a	−3.68	hsa-miR-216b	−2.56	hsa-miR-9-5p	4.17
		hsa-miR-499a-5p	−2.56	hsa-miR-24-3p	5.67

Thereafter, interaction networks of those microRNAs and the above identified genes related to atherosclerosis in the same patients, as well as of certain inflammatory genes previously related to athero-thrombosis in this pathology (24) were identified, on which several upregulated microRNAs seemed to control simultaneously the expression of various downregulated genes. Complete interaction networks are shown in Figures 5B,C.

### CD14^+^ and CD16^+^ Monocytes of RA Patients Displayed a Specific and Distinctive Activation of Intracellular Kinases

Dysfunctional intracellular signaling pathways have a pivotal role in RA, which might account for the immune-mediated chronic inflammation present in those autoimmune patients.

**Figure 7:**
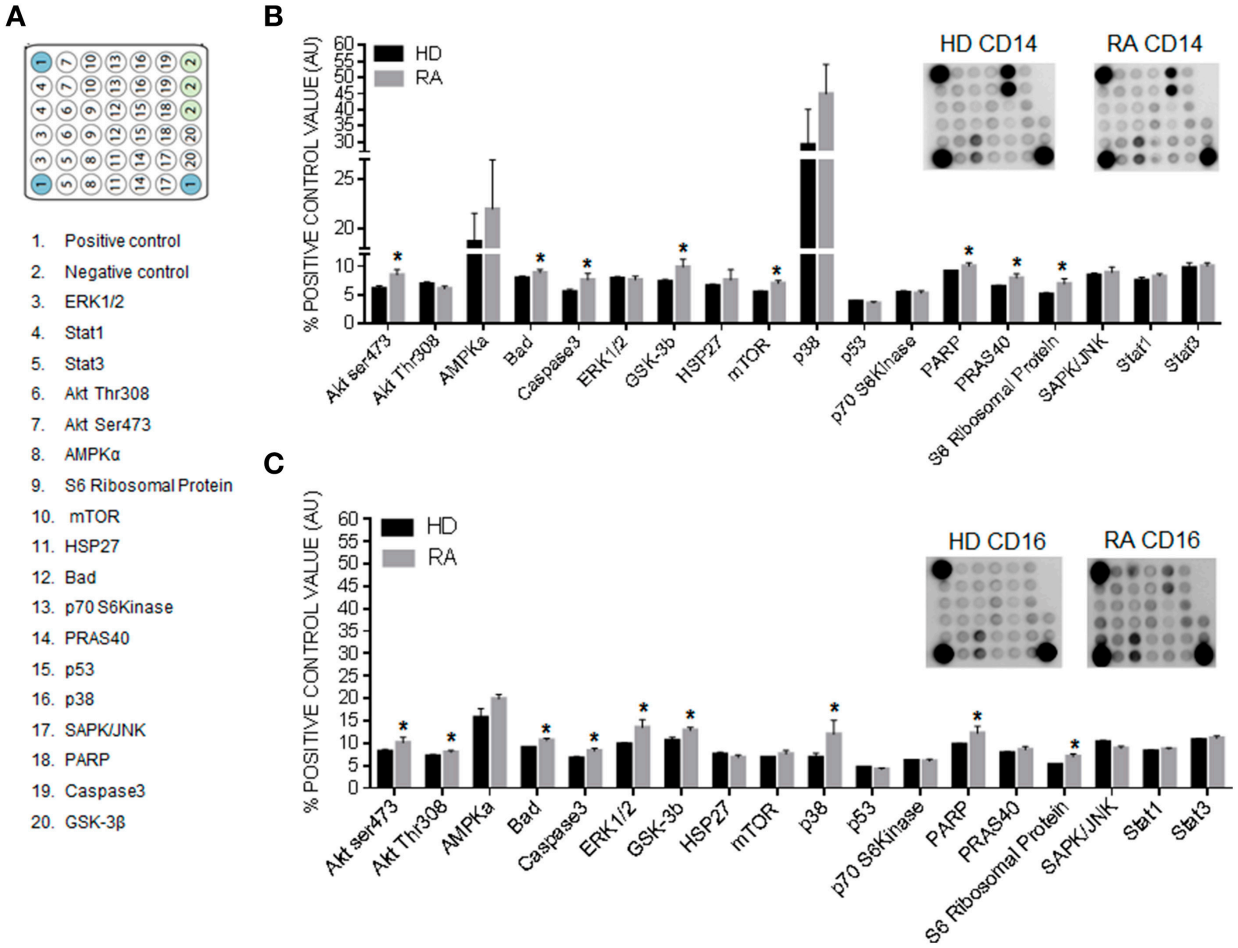
CD14^+^ and CD16^+^ monocytes of RA patients displayed a specific and distinctive activation of intracellular kinases. **(A)** Name and position in the membrane of the kinases analyzed in the PathScan intracellular signaling array. **(B)** Two representative panels of phosphorylation status of kinases using a PathScan intracellular signaling array in CD14^+^ monocytes isolated from RA and HDs. Quantification of volume intensity x area (mm2). **(C)** Two representative panels of phosphorylation status of kinases using a PathScan intracellular signaling array in CD16^+^ monocytes isolated from RA and HDs. Quantification of volume intensity x area (mm2). Data are presented as mean ± SD, *n* = 50 RA patients and 33 HDs. Paired *t*-test was performed ^*^indicates significant differences vs. HDs (*p* < 0.05).

In our hands, CD16^+^ monocytes displayed a higher number of proinflammatory kinases phosphorylated (i.e., Akt Ser473, Akt Thr308, ERK 1/2, GSK-3β, and p38) than CD14^+^ monocytes, in accordance with the increased expression of a number mRNAs coding for inflammatory and prothrombotic proteins in CD16^+^ monocyte subsets of RA patients. Yet, only two main kinases related to inflammation (including Akt Ser 473 and GSK-3 β) were found phosphorylated in CD14^+^ monocytes from RA patients (*p* < 0,05). On the other hand, six protein kinases related to survival, growth and apoptosis, (i.e., Bad, mTOR, Caspase 3, PARP, PRAS40 and S6 Ribosomal protein) were found phosphorylated in monocytes CD14^+^ from RA patients, while only 3 of those kinases were found phosphorylated in CD16^+^ monocytes (*p* < 0,05; Figure 7).

All in all, these results argue in favor of a more proinflammatory molecular profile of CD16^+^ than that of CD14^+^ monocytes in RA.

### CD14^+^ and CD16^+^ Monocyte Subsets From RA Patients Display Specific Molecular Profiles Coordinately Related to Atherosclerosis and Cardiovascular Disease

**Figure 8:**
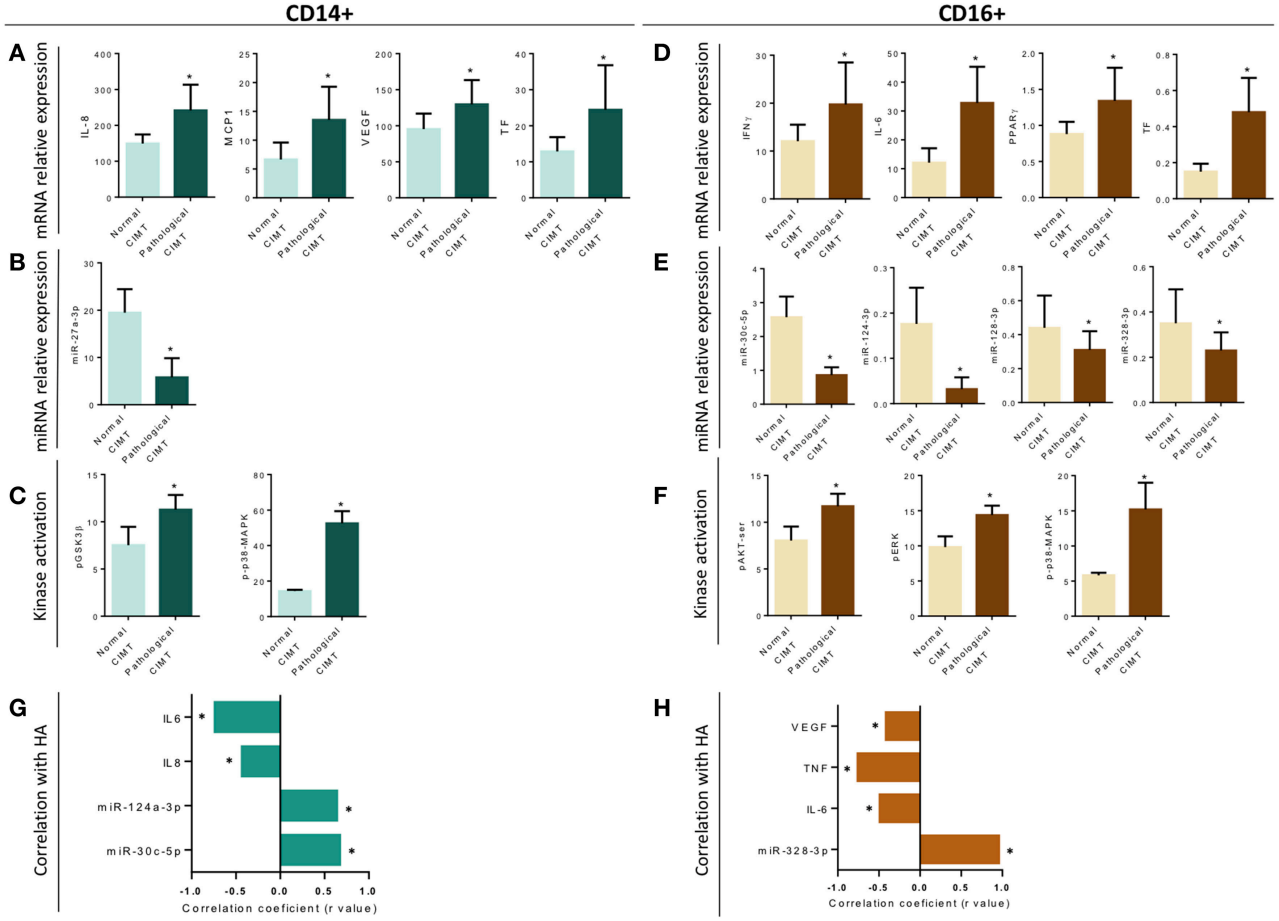
Association and correlation studies among the distinctive molecular profile of CD14^+^ and CD16^+^ monocyte subsets of RA patients and the presence of early atherosclerosis or altered microvascular endothelial function. **(A–F)** Association studies among altered mRNAs, microRNAs and distinctive kinase activation in CD14^+^ and CD16^+^ monocytes and the presence of a pathologic carotid intima-media thickness (as early atherosclerosis marker). Data are expressed as mean and standard deviation. ^*^indicates significant differences vs. normal CIMT (*p* < 0.05). **(G,H)** Spearman's rank correlations among monocyte subtype specific alterations in levels of miRNAs and microRNAs and reduced area of hyperemia after occlusion in RA patients, showing a *p* < 0,05 are indicated. Only significant correlations according to Bonferroni correction, are displayed.

Association studies (Figure 8) indicated a relationship between CIMT and increased mRNA expression levels in CD14^+^ monocytes of IL-8, MCP-1, VEGF and TF (Figure 8A), along with the reduction in the levels of microRNA-27a-3p (Figure 8B) and the increased mean phosphorylation levels of GSK3B and p38 MAPK (Figure 8C).

Accordingly, specific associations with the presence of atheroma plaques were also found in CD16^+^ monocytes, involving increased mRNA expression levels of IFNg, IL-6, PPARg, and TF (Figure 8D), reduced expression of several microRNAs (miR-30c-5p, miR-124a-3p, miR-128-3p, and miR-328-3p; Figure 8E) and increased mean phosphorylation levels of AKTser, ERK, and p38-MAPK (Figure 8F).

In parallel, impaired microvascular endothelial function, involving a reduced area of hyperemia (HA) after occlusion of blood flow in RA patients, was found also linked to a monocyte subtype-specific and significant alteration in levels of mRNAs and microRNAs (Figures 8G,H).

Correlation studies further showed a significant relationship among a number of these altered transcriptomic and intracellular signaling molecules on each monocyte subset analyzed (data not shown).

### *In vitro*, RA Serum Promoted the Differentiation of CD14^+^ Monocytes to CD16^+^ and the Coculture of Monocyte Subsets With Endothelial Cells Promoted Their Activation

**Figure 9:**
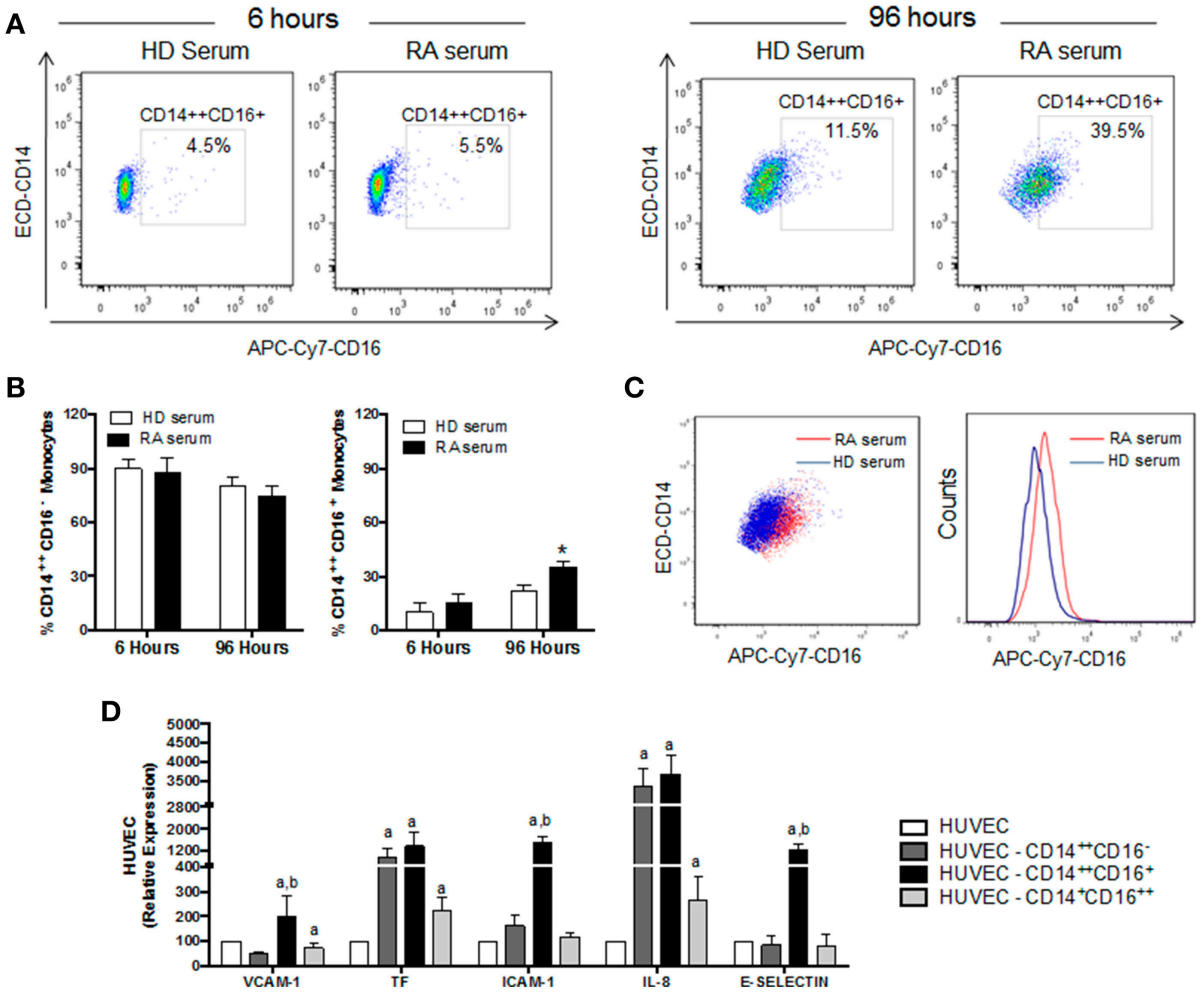
*In vitro* RA serum promoted the differentiation to CD16^+^ monocytes and the activation of endothelial cells. **(A)** Representative dot plot of intermediate monocytes of CD14^+^ monocytes isolated from HDs and treated with serum (10%) of HDs and RA patients for 6 and 96 h. **(B)** Percentage of classical monocytes (CD14^++^CD16^−^) and intermediate monocytes (CD14^++^CD16^+^) after *in vitro* treatment of CD14^+^ monocytes isolated from HDs treated with serum (10%) of HDs and RA patients for 6 and 96 h. Data represent mean ± SEM of five independent experiments. Paired *t*-test was performed ^*^indicates significant differences vs. CD14^+^ HD monocytes treated with HD serum (*p* < 0.05). **(C)** Representative overlay dot plot and histogram showing the generation of intermediate monocytes after treatment of CD14^+^ HD monocytes with serum from RA patients. **(D)** mRNA relative expression of genes involved in inflammation, cellular adhesion and coagulation in HUVECs co-cultured with the different monocyte subsets isolated from RA patients for 24 h. Data represent mean ± SEM of five independent experiments. Kruskal-Wallis test, followed by a Dunn's multiple comparison test was performed. ^a^indicates significant differences vs. HUVECs cultured alone, ^b^indicates significant differences vs. HUVECs cultured with classical RA monocytes (CD14^++^CD16^+^) HD (*p* < 0.05).

We next investigated *in vitro* the changes promoted by RA and HD serum in CD14^+^ monocytes isolated from HD. The treatment with RA serum significantly increased the expression of CD16, promoting a pronounced expansion of CD14^++^CD16^+^ monocyte subset after 96 h (*p* ≤ 0.05; Figures 9A–C).

Moreover, following co-culture with endothelial cells, cell sorted monocyte subsets isolated from RA patients, promoted a significant activation of HUVECs, as demonstrated by the increased expression of adhesion molecules (i.e., VCAM-1, ICAM-1, and E-selectin) and pro-thrombotic/pro-inflammatory mediators (i.e., TF and IL-8). Furthermore, intermediate monocytes were the major inductors of endothelial activation (Figure 9D).

## Discussion

In the present study we provide evidence, in a large cohort of RA patients, that monocyte subset distribution is skewed to a more “pro-inflammatory” profile, with elevated frequency of intermediate monocytes (CD14^++^CD16^+^), which were related to the autoimmune and inflammatory profile, the altered microvascular function, the occurrence of early atherosclerosis, and the presence of an increased score of cardiovascular disease risk in this autoimmune disorder.

Besides, we have characterized the molecular profile of CD14^+^ and CD16^+^ monocyte subsets in RA, including the identification of their genetic and epigenetic profiles related to atherogenesis and cardiovascular risk and the activated intracellular pathways that modulate those proinflammatory and prothrombotic alterations.

Monocytes and monocyte-derived macrophages are involved in all stages of atherogenesis, from initiation and progression, to destabilization and rupture of atherosclerotic plaques, with potential harmful outcome (25–27). The CD16^+^ subclass has been previously shown to be pro-inflammatory, being proportionally elevated in diseases with underlying inflammation (28, 29). Moreover, in CVDs such as acute coronary syndrome, monocyte subset distribution was skewed to an increased proportion of CD16-positive cells when compared with HD. Indeed, a large study evaluating monocyte subset distribution as an outcome predictor in more than 900 stable CAD patients established the intermediate monocyte population as an independent predictor of cardiovascular events (30).

Accordingly, in our RA cohort, the increased percentage of intermediate monocytes was associated with a pathological increase in carotid intima media thickness. Besides, the percentage of this monocyte subset correlated to an increased score of cardiovascular disease risk.

Currently, little is known about the importance of non-classical monocytes and their role in atherosclerotic development and CVD in the setting of RA. It has been shown that in response to inflammation, vascular damage or infection, chemoattractant factors are released from either damaged tissue, endothelial cells, or recruited immune cells, which attract these so-called patrolling monocytes -due to their capacity of extravasation from vascular endothelium after tissue injury-. Then, these monocytes are recruited to sites of vascular injury or enter areas of inflammation -i.e., arthritic joints, atherosclerotic plaques or nephritic kidneys (31).

Both classical and non-classical subsets can enter atherosclerotic plaques in mice (32). Moreover, the number of non-classical monocytes is inversely correlated with high-density lipoprotein cholesterol levels in hypercholesterolemic patients and is associated with increased APO-E expression, a factor linked to higher plasma cholesterol (33), and thus, a potential contributor to atherosclerosis development.

Hence, in our RA cohort, non-classical monocytes showed, in parallel to intermediate monocytes, increased expression of pro-thrombotic and inflammatory parameters (i.e., TF, TNFα, or IL-6), which were further associated to the presence of atheroma plaques and to the microvascular endothelial dysfunction exhibited by RA patients. Moreover, we identified a relationship among the percentage of this monocyte subset and the presence of CV risk factors such as hypertension and hypertriglyceridemia.

That overall data argues in favor of the presence of some shared profiles among intermediate and non-classic monocytes related to the enhanced CV risk present in RA patients.

As above mentioned, even although previous studies attributed a key role of intermediate monocytes to vascular involvement in many CVD (30), we have found a number of altered parameters in non-classical monocytes also related to increased CV risk, which might indicate that both CD16^+^ monocyte subsets may share molecular alterations related to this comorbidity in the setting of RA. Thus, to adequately characterize the molecular profile of distinct monocyte subsets involved in CVD in the setting of RA, we developed transcriptomic and intracellular signaling comparative studies between the global CD16^+^ monocytes population and the classical CD14^+^ monocytes subsets.

The detailed molecular characterization of CD16^+^ monocyte subsets supported the notion of their atherothrombotic potential, so that we identified a global pro-atherogenic gene profile involving the overexpression of a number of cytokines, chemokines, and genes implicated in migration, lipid metabolism and cell adhesion. This gene profile differed from that displayed by CD14^+^ monocytes, suggesting different functionalities of these cells related to CVD.

MicroRNAs (microRNAs) are small noncoding RNAs that have an essential role in the regulation of genome expression at the posttranscriptional levels, playing relevant roles in autoimmunity and CVD. We previously identified, in monocytes from patients with APS and SLE, several deregulated microRNAs, which modulated the prothrombotic status of those autoimmune patients (34). We here investigated the profile of microRNAs in CD14^+^ and CD16^+^ monocyte subsets and their relationship with the inflammatory and prothrombotic shape of patients with RA. Our data demonstrated, for the first time, the presence of a distinctive and specific microRNA profile on CD16^+^ monocytes, characterized by a higher number of different deregulated microRNAs than CD14^+^ monocytes. The functional classification of those microRNAs demonstrated that the majority of them have potential targets involved in inflammation, immunity and thrombotic processes. Therefore, the altered levels of those microRNAs might be associated with the pro-atherothrombotic profile of RA patients. That hypothesis was supported by the identified relationship among the presence of atheroma plaques and the altered expression of specific microRNAs on each monocyte subset.

In addition, the integrated analysis of validated microRNAs and the altered genes identified in the PCR array of atherosclerosis demonstrated the presence of a more complex network on CD16^+^ monocytes, on which several reduced microRNAs seem to control simultaneously the expression of various over-expressed genes. Thus, we have identified novel and specific microRNA–mRNA regulatory networks in monocyte subsets related to CVD in patients with RA.

The cellular activities observed in monocytes, associated to increased production of cytokines, thrombotic factors, mediators of lipid metabolism, and adhesion molecules, are determined by the specific signaling pathways that are activated. It has been shown that proteins from the synovial tissue of RA patients are phosphorylated by intracellular tyrosine kinases, underlying the importance of tyrosine kinases in the pathogenesis of this autoimmune disorder (35). Yet, this is the first study identifying the activation status of main signaling pathways linked to RA pathogenesis in monocyte subsets. The use of an array allowed us to demonstrate that CD16^+^ monocytes displayed a higher number of activated intracellular kinases than CD14^+^ monocytes, pointing to a more activated status, and thus to enlarged inflammatory and prothrombotic profiles. Hence, in our RA cohort, these activated profiles were specifically associated in both monocyte subsets with either, the altered expression of a number of genes related to atherosclerosis, and with the presence of atheroma plaques, further supporting the involvement of these monocyte subsets in the enlarged CV risk displayed by RA patients.

The heightened risk of CVD is associated to the incidence of early endothelial dysfunction in both the microvasculature and microvasculature, even in case atherosclerosis is not detectable. Macrovascular endothelial dysfunction is recognized as the main contributor to atherogenesis of the large arteries. Yet, in many typical CVDs, microvascular endothelial dysfunction is also considered a warning signal that heralds the development of artery atherosclerosis and cardiovascular disease (36).

Accelerated atherosclerosis and increased risk of CVD accompanying RA is linked to endothelial activation and dysfunction. We recently reported the occurrence of endothelial dysfunction in a cohort of RA patients, involving both, altered microvascular function and increased activation of endothelial cells, which was further prevented by *in vivo* treatment with tocilizumab (22).

Accordingly, in the present study a significant alteration in the microvascular endothelial function was noticed, which was further associated with the increased percentage of inflammatory monocyte subsets, as well as with the global molecular profile of CD16^+^ monocytes (in terms of gene, microRNA and intracellular signaling alterations). These results were confirmed on *in vitro* studies, on which endothelial cells co-cultured with monocyte subsets isolated of RA patients promoted their activation, including the over expression of cell-adhesion molecules, interleukins and the main inductor of coagulation, tissue factor. Our *in vitro* studies further demonstrated that the autoimmune and inflammatory mediators present in the plasma of RA patients directly influence the generation of intermediate monocytes, which in turn overexpress inflammatory molecules and activate the endothelium, thus inducing a loop of inflammation that favor their heightened CV risk.

Our study is not without limitations and thus some aspects need to be interpreted with caution. The first cohort of RA patients analyzed significantly differed in age in relation to healthy donors. Moreover, this is at an age where there is a lot of hormonal flux in women, and this potentially may contribute to some of the differences seen in RA vs. HD. In recent studies, it has been demonstrated that several female hormonal factors are associated with RA development (37). Estrogens, at levels reached at the periovulatory to pregnancy states, stimulate B cells and the Th2 response and sustain the survival of auto-reactive T and B cells (38, 39). However, during menopause, the drop in ovarian function and thus in circulating levels of estrogens is associated with increased production of pro-inflammatory cytokines (i.e., IL-6, TNF-α, and IL-1β) (40–42).

With these premises, we analyzed in our RA cohorts the influence of age as well as of the pre- or post-menopausal status, on the percentage monocytes subsets, as well as on parameters related to the activity of the disease, autoantibody profile, and the expression levels of inflammatory mediators regulated by hormonal factors. We found that nor the age nor the menopausal status acted as a confounding variable among RA patients in any of those parameters, indicating that these alterations might be related to intrinsic elements associated to this autoimmune disorder.

## Conclusions

All in all, our overall data suggest that the generation of inflammatory monocytes is associated to the autoimmune/inflammatory response that mediates RA. These monocyte subsets, -which display specific and distinctive molecular signatures- might promote endothelial dysfunction and in turn, the progression of atherosclerosis through a finely regulated process driving CVD development in RA.

## Ethics Statement

This study was carried out in accordance with the recommendations of the American College of Rheumatology/European League Against Rheumatism criteria for the classification of RA and the ethical committees of each of the participating centers in the PRECISESADs project with written informed consent from all subjects. All subjects gave written informed consent in accordance with the Declaration of Helsinki. The protocol was approved by the Ethical Committees of each of the participating centers in the PRECISESADs project.

## Author Contributions

PR-L, NB, EC-E, MEA-R, and CL-P formed the hypothesis, directed and coordinated the project, designed the experiments, analyzed the data and wrote the manuscript. The members of the PRECISESADS Clinical Consortium, RO-C, AE-C, LP-S, JC-G, and PF followed up with patients and contributed useful discussion and suggestions. MA-A, AMP-T, AI-C, ML-T and CP-S developed the *in vivo* assays, performed the experiments and solved technical problems. The members of the PRECISESADS Flow Cytometry Study Group, CJ, IdlR, and YJ-G were involved in flow cytometry methods, statistical analysis and discussing related results.

## Consortium

The members of the PRECISESADS Flow Cytometry Study Group are CJ, Marañón C, Le Lann L, Varela N, Muchmore B, Dufour A, Alvarez, Carlo Montserrat Chizzolini C, De Langhe E, NB, CL-P, Gerl V, De Groof A, Ducreux J, Trombetta E, Li T, Alvarez-Errico D, Rao S, and Pers JO.

The members of Precisesads Clinical Consortium are Beretta L, AguilarQuesada R, Aguirre-Zamorano MA, Callejas Rubio JL,Castro-Villegas MC, Cervera R, Chizzolini C, Collantes E, Cornec D, De Langhe E, Devauchelle-Pensec V, AE-C, Espinosa G, Fernández Roldán MC, Gomes Anjos T, Hiepe F, Jiménez Moleón I, Jousse-Joulin S, Lauwerys B, López-Berrio A, Lories R, Marovac J, Meroni PL, Miranda B, Navarro-Linares H, Ortega-Castro R, Ortego N, Pers JO, Ramón Garrido E, Raya E, Ríos Fernández R, Rodríguez-Pintó I, and Saraux A.

## Conflict of Interest Statement

The authors declare that the research was conducted in the absence of any commercial or financial relationships that could be construed as a potential conflict of interest.
