# Supplementary material for: Molecular Characterization of Monocyte Subsets Reveals Specific and Distinctive Molecular Signatures Associated With Cardiovascular Disease in Rheumatoid Arthritis
Source: Front Immunol. 2019 May 21;10:1111. doi: 10.3389/fimmu.2019.01111 (PMC6536567; doi:10.3389/fimmu.2019.01111)
Supplement: Supplementary file 1 [file Data_Sheet_1.pdf]

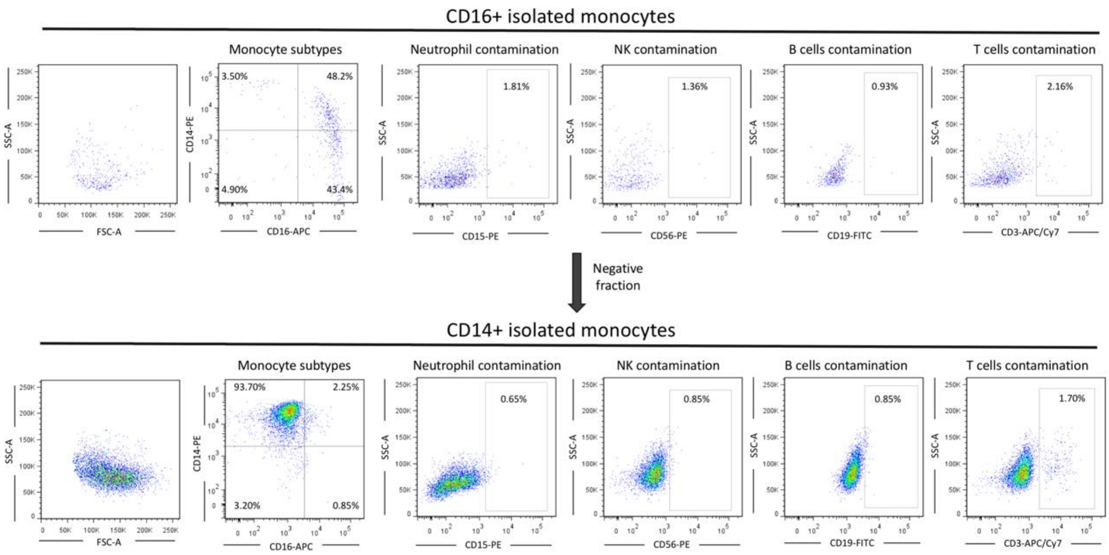

Purity of the CD16⁺ and the CD14⁺ isolated cells through positive immunomagnetic selection. Dot plots showing the purity of isolated populations. To test the contamination of neutrophils, NK, B cells and T cells PE anti-human CD15, PE anti-human CD56, FITC anti-human CD19 and APC/Cy7 anti-human CD3 were used. Percentage of $CD14^+$ and $CD16^+$ cells was determined by PE anti-human CD14, APC anti-human CD16.
