# Supplementary figures and images for: Molecular Characterization of Monocyte Subsets Reveals Specific and Distinctive Molecular Signatures Associated With Cardiovascular Disease in Rheumatoid Arthritis
Source: Front Immunol. 2019 May 21;10:1111. doi: 10.3389/fimmu.2019.01111 (PMC6536567; doi:10.3389/fimmu.2019.01111)

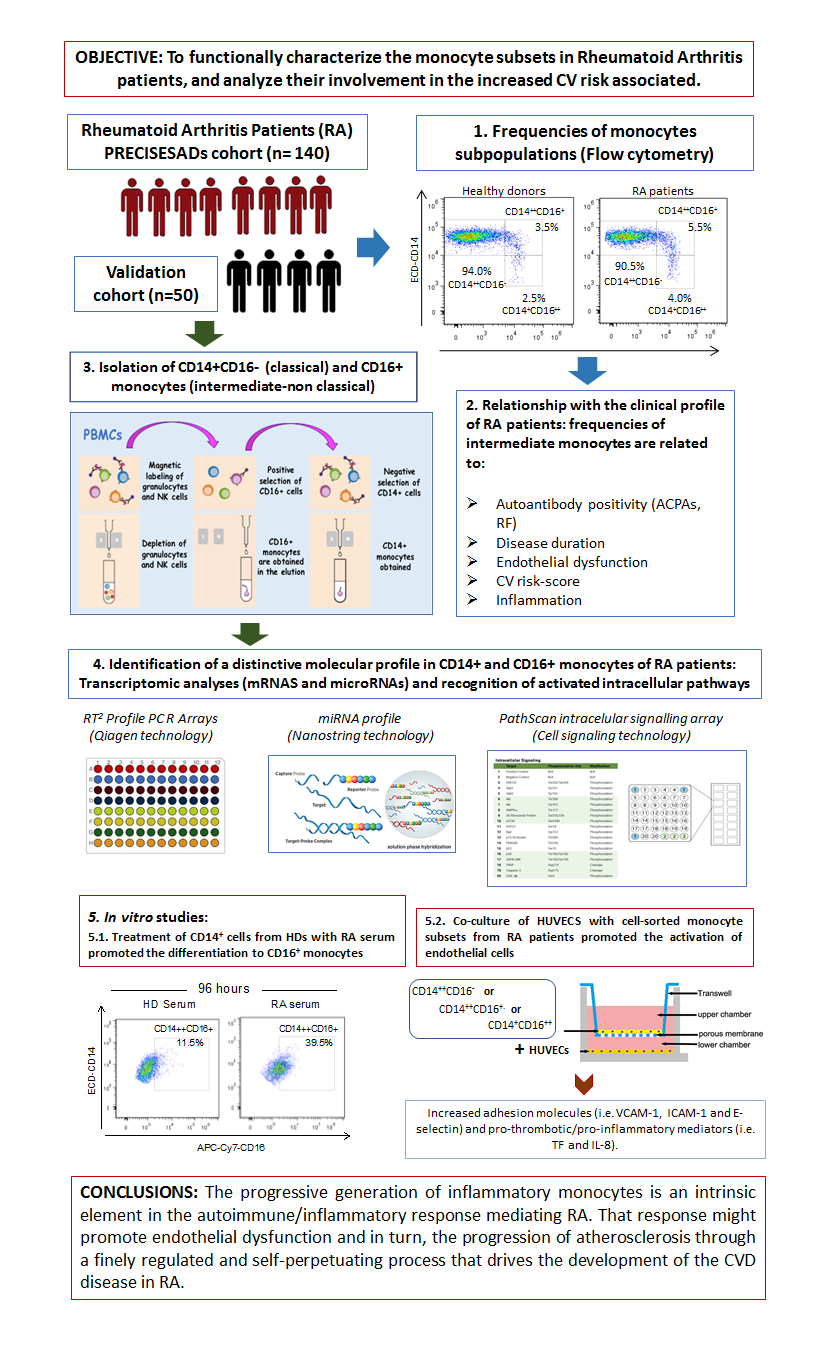

Supplement: Supplementary file 2 [file Image_1.TIF]
